# Effects of globus pallidus internus deep brain stimulation on freezing of gait in Parkinson's disease: a prospective short-term single-arm study

**DOI:** 10.3389/fnagi.2026.1848040

**Published:** 2026-09-28

**Authors:** Pingchen Zhang, Linghao Cao, Tengjiao Wang, Dianyou Li, Shengdi Chen, Jun Liu, Hua He, Haiyan Zhou

**Affiliations:** 1 Department of Neurology and Institute of Neurology, Ruijin Hospital, Shanghai Jiao Tong University School of Medicine, Shanghai, China; 2 Department of Neurology, Ruijin Hospital Luwan Branch, Shanghai Jiao Tong University School of Medicine, Shanghai, China; 3 Joint Research Center for Medicine, Science and Technology, Translational Medicine Research Center, Naval Medical University, Shanghai, China; 4 Department of Neurosurgery, Ruijin Hospital, Shanghai Jiao Tong University School of Medicine, Shanghai, China; 5 Department of Neurosurgery, Shanghai Eastern Hepatobiliary Surgery Hospital, Shanghai, China

**Keywords:** axial symptoms, deep brain stimulation, freezing of gait, globus pallidus interna, Parkinson's disease, prospective study

## Abstract

**Background:**

Freezing of gait (FoG) is a debilitating symptom in Parkinson's disease (PD), often resistant to medication. Deep brain stimulation (DBS) has emerged as an effective treatment for advanced PD, while its effect on FoG remains inconsistent. Specifically, the effect of globus pallidus internus deep brain stimulation (GPi-DBS) against FoG is inconclusive, and relevant studies are scarce.

**Objective:**

To describe the post-operative effect of GPi-DBS on FoG and identify pre-operative predictors of improvement.

**Methods:**

Forty-six PD patients undergoing GPi-DBS were assessed pre-operatively and at short-term follow-up (a median of 11 months) using the Gait and Falls Questionnaire (GFQ) and a comprehensive Timed Up and Go (TUG) test that quantified five FoG-related TUG indices. The primary outcome was the within-individual change in TUG total time, computed as the pre-operative med-off value minus the DBS-on med-off value. The secondary outcomes were changes of pre-operative med-off vs. med-on (the acute levodopa response) and pre-operative med-off vs. DBS-off med-off (a within-subject negative control for disease progression). Pre-operative predictors of FoG were assessed to predict the within-individual change in the TUG indices by performing forward stepwise Akaike Information Criterion (AIC) regression analysis.

**Results:**

At short-term follow-up, FoG measures (the TUG-derived indices and the self-reported GFQ) improved after GPi-DBS. TUG total time decreased by a median of 5.0 s (95% CI 1.5–14.5; FDR = 0.003), and the longest FoG duration by 1.0 s (95% CI 0–3.5; FDR = 0.030); the other three TUG indices improved in the same direction but did not survive correction (FDR 0.064–0.080). Additionally, GFQ total score decreased from a baseline median of 24 to 17 (Hodges–Lehmann median difference 4.0 points; 95% CI 0–8; *n* = 46; *p* = 0.036). In the forward-stepwise AIC regression (*n* = 40), the pre-operative axial levodopa response was the only pre-operative variable retained for the TUG changes; among the four motor components, only the DBS- related change in the axial subscore was significantly correlated with TUG improvement (Spearman ρ = 0.431, 95% CI 0.138–0.654, *p* = 0.006).

**Conclusions:**

Within this single-arm cohort, FoG measures improved over short-term follow-up after GPi-DBS, and greater pre-operative axial levodopa-responsiveness was associated with greater TUG improvement.

## Introduction

Parkinson's disease (PD) is one of the most common neurodegenerative disorders, manifesting with motor and non-motor symptoms. One of the most disabling symptoms at the advanced stage is freezing of gait (FoG), which is characterized by the sudden and temporary inability to initiate or continue walking despite the intention to walk (Nutt et al., 2011). FoG can occur at any stage of PD, but it is more common in advanced stages of the disease, affecting up to 60% of patients (Zhang et al., 2021).

FoG has a significant impact on the quality of life of PD patients, leading to falls, injuries, hospitalization, social isolation, and depression (Cong et al., 2022; Walton et al., 2015). Current management of FoG faces dual challenges. First, therapeutic options are limited in efficacy. Levodopa lessens freezing in some patients, but a substantial proportion of freezing is not dopamine-responsive (McKay et al., 2019). Second, accurate detection of FoG remains technically challenging. While wearable devices show moderate reliability in detecting prolonged FoG episodes in controlled laboratory settings, brief or sporadic FoG events (e.g., freezing during turns or gait initiation) in real-world environments are difficult to capture with high precision (Mancini et al., 2021). These detection limitations not only complicate therapeutic evaluation but also hinder the development of targeted interventions.

Deep brain stimulation (DBS) has emerged as an effective treatment for the motor symptoms of PD, including tremors, rigidity, and bradykinesia, as well as motor complications. DBS involves the implantation of electrodes into specific regions of the brain, which are then stimulated to modulate neuronal activity. The most commonly targeted regions for DBS in PD are the subthalamic nucleus (STN) and the globus pallidus internus (GPi) (Deuschl et al., 2022).

A few studies have investigated the effect of DBS on FoG in PD with mixed results. While some studies have reported significant improvements in FoG following STN-DBS (Barbe et al., 2020; Gavriliuc et al., 2020; Kim et al., 2019), others have found no significant effect in certain patients (Mei et al., 2020), especially for those with improper activated cortico-subthalamic network recruitment (Temiz et al., 2022). In contrast, the existing research on GPi-DBS for the prevalence of gait disturbances is relatively scarce compared to STN (Au et al., 2021), necessitating further investigation. Globally, GPi-DBS has been empirically proposed as a better target for severe gait difficulties than STN (Mei et al., 2020). The GPi is a key component of the basal ganglia. It receives input from the striatum and sends inhibitory signals to the thalamus, which in turn modulates cortical activity (Lanciego et al., 2012). GPi has been shown to have strong connections to primary motor cortex, supplementary motor area (SMA), and motor thalamus, as abnormal GPi activity alters downstream motor pathways, exacerbating gait disturbances (DiRisio et al., 2023).

While another alternative target pedunculopontine nucleus (PPN) has been explored for refractory FoG, outcomes remain inconsistent due to its diffuse connectivity and technical challenges (e.g., optimal stimulation parameters, proximity to critical vasculature) (Bourilhon et al., 2022; Lin et al., 2023). PPN-DBS also carries higher risks of dysarthria and paresthesia, positioning GPi as a more reliable target within established DBS paradigms.

Here, we designed a prospective clinical study aiming to investigate the effect of GPi-DBS on FoG and identify potential predictable factors related to a good response. In addition to Movement Disorder Society (MDS)-Unified Parkinson's Disease Rating Scale (MDS-UPDRS), we conducted a comprehensive evaluation of the severity of FoG using various standardized assessment tools, such as the Gait and Falls Questionnaire (GFQ) (Giladi et al., 2000) for subjective complaints from PD patients and the timed up and go (TUG) test (Podsiadlo and Richardson, 1991) for quantification analysis. This single-arm prospective study aimed to (1) evaluate the effect of GPi-DBS on FoG in PD patients over short-term follow-up; and (2) identify pre-operative predictors of post-operative FoG improvement assessed by 28 pre-operative clinical characteristics, including MDS-UPDRS III constructs and demographic or clinical variables.

## Methods

### Study design and participants

Forty-eight patients were recruited prospectively from the Departments of Neurology and Functional Neurosurgery at Ruijin Hospital affiliated to Shanghai Jiaotong University School of Medicine (Shanghai, China) from November 2017 to January 2019, using the following inclusion criteria: (1) idiopathic PD, according to the MDS Parkinson's Disease Society Diagnostic Criteria (Postuma et al., 2015); (2) bilateral GPi-DBS implant according to a careful consideration of patients' symptom profiles (e.g., existence and severity of dyskinesia, dystonia, axial symptoms, or cognitive and mood problems); (3) onset of PD at or before 70 years of age; and (4) disease duration of more than 3 years at the time of surgery. Patients with comorbidities affecting gait (e.g., severe osteoarthritis, peripheral neuropathy, stroke history) were excluded at the time of the study.

GPi was preferred over the STN when patients had prominent levodopa-induced dyskinesia or dystonia, more pronounced axial or gait involvement, or borderline cognitive or mood profiles, for which GPi offers a wider therapeutic window and a lower risk of stimulation-induced axial or neuropsychiatric worsening; the STN was preferred when a large reduction of dopaminergic medication was an important consideration, consistent with evidence that STN stimulation permits greater medication reduction whereas target choice also rests on non-motor and axial considerations (Follett et al., 2010; Odekerken et al., 2013). All patients underwent GPi-DBS based on the institutional guideline for DBS surgery. Stereotactic implantation of bilateral GPi-DBS (Medtronic, Activa 3387) with implantable pulse generators (Medtronic, Activa) was performed in general anesthesia. Post-operative CT-MRI fusion confirmed that electrodes were targeted at the posteroventral GPi with stimulation contacts within 2 mm of this target considered optimal (Holland et al., 2022), approximately 2–4 mm anterior to the midpoint of the anterior commissure–posterior commissure (AC-PC) line, 18–22 mm lateral to the AC-PC line, and 2–4 mm below the AC-PC line as established in our prior work (Lai et al., 2022). The stimulation settings and medication were adjusted individually to maintain optimal clinical condition. The study report was conducted in accordance with the Declaration of Helsinki. All participants provided written informed consent. The study protocol (No. 2017–80) was approved by the Ruijin Hospital Ethics Committee of Shanghai Jiao Tong University School of Medicine (Shanghai, China) and is publicly registered in clinicaltrials.gov (NCT03227250).

### Outcome measures

All patients underwent standardized evaluation pre-operatively and post-operatively, similar to our previous study (Shen et al., 2023). All participants were on levodopa-containing regimens. They provided information on disease history, medication usage for the calculation of levodopa equivalent daily dose (LEDD) (Tomlinson et al., 2010), Mini Mental State Examination (MMSE) for cognition (Skorvanek et al., 2018), and GFQ for self-reported FoG severity (Giladi et al., 2000). The one commonly used classification of motor subtypes was calculated and the PD patients were divided into three categories: tremor-dominant (TD) patients (ratio ≥1.15), postural instability and gait difficulty (PIGD) patients (ratio ≤ 0.90), and indeterminate (ID) patients (ratios >0.9 and < 1.15) (Stebbins et al., 2013).

The Movement Disorder Society (MDS)-Unified Parkinson's Disease Rating Scale (MDS- UPDRS) (Goetz et al., 2007), the Hoehn and Yahr staging (H-Y) (Goetz et al., 2004), and TUG test (Podsiadlo and Richardson, 1991) were examined. The TUG test assesses mobility by measuring the time taken to stand from a chair, walk 3 m, turn, and return back to the chair. Change in TUG total time was designated as the primary outcome measure, computed within each individual as the pre-operative med-off value minus the DBS-on med-off value, so that positive values denote improvement; this contrast matches the med-off state across time points and therefore isolates the effect of stimulation from that of medication. To granularly evaluate the FoG severity, the FoG-related indices in the TUG test were subdivided into four other categories: FoG total time in TUG test, FoG episodes, proportion of FoG in TUG test, and the longest FoG duration. To minimize evaluation bias, all video recordings of MDS-UPDRS III (excluding rigidity) and TUG tests were performed by a single specialist (Haiyan Zhou) and subsequently de-identified through removal of temporal/condition identifiers. These anonymized videos were independently scored by two certified movement disorder specialists (Pingchen Zhang and Linghao Cao) who were blinded to surgical status (pre/post-operative), DBS activation state, and clinical management history, with discrepancies resolved by a third arbitrator (Dianyou Li). Due to the technical requirement for tactile assessment, rigidity was evaluated unblinded by the treating clinician (Haiyan Zhou), with final MDS-UPDRS III scores reported as composite scores.

Pre-operatively, a 12-h overnight withdrawal of dopaminergic medications was required to perform off-medication (pre-operative med-off) motor assessments. After taking 150% of their morning levodopa equivalent dose as a suprathreshold dose of levodopa preparation and waiting for approximately 60 min to reach the best motor response state (Zeng et al., 2024), the patients underwent the on-medication motor assessments (pre-operative med-on). At post-operative follow-up, all patients were assessed under two conditions post-operatively: DBS- on medication off (DBS-on med-off) and DBS-off medication off (DBS-off med-off). DBS-on med-off evaluations were performed following a 12-h overnight dopaminergic medication cessation and kept DBS-on. DBS-off med-off evaluations were performed subsequently after turning stimulation off for 1 h.

The primary contrast was pre-operative med-off vs. DBS-on med-off, because matching the med-off state isolates the effect of stimulation from that of medication. Two secondary, descriptive contrasts were pre-operative med-off vs. med-on (the acute levodopa response) and pre-operative med-off vs. DBS-off med-off (a within-subject negative control for disease progression, since the untreated off-state was not expected to change materially over the short-term follow-up); the remaining contrasts were reported for completeness only. We examined whether 28 pre-operative variables predicted the within-individual change in the TUG indices from the pre-operative med-off to the DBS-on med-off condition. For this, we extracted four MDS-UPDRS III motor components: tremor (items 3.15–3.18), rigidity (item 3.3), bradykinesia (items 3.2, 3.4–3.8, 3.14), and axial symptoms (items 3.1, 3.9–3.13). For each component, we calculated two metrics: (1) the difference between pre-operative med-off and med-on scores (the acute levodopa response), and (2) the percentage improvement [(med-off score–med-on score)/med-off score × 100%].

### Statistical analysis

A power analysis was conducted to determine the sample size, based on prior data from Huang et al. (2011) on the minimal detectable change (MDC) of the TUG test in PD patients (MDC = 3.5 s, baseline SD = 2.9 s). Using these values, the effect size (Cohen's d) was calculated as 1.2. With a significance level (α) of 0.05 and desired power (1–β) of 0.95, G^*^Power 3.1 analysis indicated a minimum sample size of 12 patients. To address potential attrition and enhance robustness, 48 patients were initially enrolled; 46 were finally analyzed, exceeding the estimated sample size requirement and ensuring the sample size required for the primary TUG contrast.

Demographic variables, motor variables at baseline and follow-up were described. Continuous variables were tested for normality with the Shapiro–Wilk test; normal data were expressed as mean ± standard deviation and non-normal data as median (lower quartile, upper quartile), and categorical variables were presented as percentages and numbers. All statistical analyses and figures were produced using Python 3.10 (Python Software Foundation, Wilmington, DE, USA).

Because most continuous variables did not meet the assumption of normality (already tested above), pre- and post-operative scores were compared using paired Wilcoxon signed-rank tests; the within-individual difference is reported as the Hodges–Lehmann median with a distribution-free 95% confidence interval. When we encountered multiple comparisons, *p* values were adjusted by FDR (Benjamini and Hochberg, 1995). For multiplicity we used Benjamini–Hochberg FDR within pre-specified families: the six pairwise contrasts of each TUG index; the 10 MDS-UPDRS III item comparisons; the 16 GFQ item comparisons; the five regression outcomes; and the five axial-component correlations; the number of comparisons is stated in each table footnote.

To identify pre-operative factors impacting the within-individual TUG change (pre-operative med-off to DBS-on med-off) while avoiding collinearity and balancing model complexity with goodness-of-fit, we implemented a forward stepwise Akaike Information Criterion (AIC) regression model (Yamashita et al., 2007). This approach included 28 pre-operative variables: eight demographic or clinical variables (sex, age at onset, disease duration, MMSE, LEDD, med-off and med-on Hoehn–Yahr, and the TD/PIGD ratio), and, for each of five MDS-UPDRS III constructs (total score, tremor, rigidity, bradykinesia and axial), four derived forms—med-off, med-on, the med-off minus med-on difference (the acute levodopa response), and the percentage improvement—giving 5 × 4 = 20 variables (28 variables in total).

Two within-individual axial differences are distinguished. The axial levodopa response (pre-operative med-off minus med-on) was evaluated as a candidate predictor in the AIC model. Separately, to test which motor component tracked gait improvement, we correlated the DBS-related change in each MDS-UPDRS III motor subscore (pre-operative med-off minus DBS-on med-off) with the corresponding within-individual TUG change (pre-operative med-off minus DBS-on med-off) using Spearman's rank correlation with 95% confidence intervals.

Outcomes were analyzed in pre-specified sets: the whole cohort (*n* = 46) for the questionnaire outcomes (GFQ) and the MDS-UPDRS III; the 34 participants with complete TUG in all four conditions for the four-condition TUG comparison; and the 40 participants with paired pre-operative med-off and DBS-on med-off TUG for the regression of the TUG change and the component-specificity correlation. The reason for the discrepancy in the number of participants across different sets is that 12 participants could not complete it under every required condition. They were either unwilling to endure prolonged off state, especially combined medication-off and stimulation-off (“double-off”) state or unable to complete the full walking course due to severe gait disturbance—so a few assessment time points were missing and complete TUG data were not available for every patient. FoG status was assessed with MDS-UPDRS II item 13 (Goetz et al., 2007); participants without baseline FoG were retained for cohort description and for the analysis of new-onset FoG.

## Results

### Characteristics for the entire cohort

During follow-up, one patient was lost to follow-up and one withdrew consent, resulting in a final cohort of 46 Parkinson's disease patients who completed bilateral GPi-DBS implantation and assessment. This pre-dominantly male cohort (54%, *n* = 25) had a mean age of onset at 50.5 ± 8.1 years and disease duration of 10.89 ± 4.14 years at surgery. Follow-up was short-term, with reassessment obtained at a median of 11 months (range 6–34; mean 12.2 ± 7.4 months). All patients underwent FoG evaluation using MDS-UPDRS II item 13 (Goetz et al., 2007), revealing 33 (72%) were FoG-positive at baseline—subclassified by their examiner-rated freezing response (MDS-UPDRS III item 3.11) to an acute suprathreshold levodopa challenge into levodopa-responsive (OFF-FoG, item 3.11 resolved to 0 on levodopa, *n* = 23), partially levodopa-responsive (item 3.11 reduced but not resolved, *n* = 7), and levodopa- unresponsive (item 3.11 unchanged or worse, *n* = 3) freezing (Supplementary Table 1), with no ON-FoG cases (Espay et al., 2012). The responsive and unresponsive poles correspond to the classical off- vs. on-state subtyping of freezing by its levodopa response (Schaafsma et al., 2003) and to the persistence of freezing after an acute levodopa challenge (McKay et al., 2019); the intermediate partially-responsive tier is an operational extension introduced here to capture graded response and has not itself been externally validated. Phenotypically, baseline distribution included nine tremor-dominant, seven indeterminate, and 30 postural instability/gait difficulty (PIGD) cases, progressing to 74% PIGD pre-dominance at follow-up.

At follow-up, medication burden fell by a median of 210 mg (95% CI 138–278; *p* < 0.001) from a pre-operative mean LEDD of 823.8 ± 278.0 mg (Table 1). (Consistent with our pre-specified approach for all within-individual comparisons, the reduction is summarized via the Hodges–Lehmann estimator regardless of this variable's own distribution, whereas the baseline and follow-up levels themselves are summarized as mean ± SD because each independently meets normality.) The acute pre-operative levodopa challenge (med-off to med-on) produced a motor improvement of 57.28 ± 12.85%. Chronic GPi-DBS stimulation produced a median motor improvement of 31.82% (21.67, 45.20) from the pre-operative med-off state to the DBS-on med-off state (Table 1). Post-operative stimulation parameters (voltage, pulse width, frequency) are given in Table 1. Based on MDS-UPDRS II item 2.13, self-reported FoG severity improved significantly over the follow-up among the 33 patients with FoG at baseline (Wilcoxon signed-rank *p* = 0.022): seven (21%) became FoG-free (item 2.13 = 0) and eight improved, while 12 were unchanged and six worsened; only two of the 13 baseline FoG-negative patients (15%) developed new-onset FoG. Consistent with the objective TUG and questionnaire findings, this indicates a reduction in FoG severity among affected patients after GPi-DBS; the binary FoG prevalence (33 vs. 28 of 46) did not change significantly (McNemar *p* = 0.180), reflecting these few new-onset cases (Supplementary Table 2). More detailed demographic characteristics could be found in Table 1.

**Table 1:** Cohort characteristics at baseline and follow-up (*n* = 46).

Characteristic	Baseline	At follow-up
Male, *n* (%)	25 (54%)	—
Age at onset, y	50.5 ± 8.1	—
Disease duration at surgery, y	10.89 ± 4.14	—
Follow-up, months	—	11 (range 6–34; mean 12.2 ± 7.4)
Hoehn–Yahr (2.0/2.5/3.0/4.0)	5/19/9/13	6/16/12/12
Phenotype (TD/ID/PIGD)	9/7/30	8/4/34
MMSE	28.0 (27.0, 29.0)	–
FoGQ (GFQ items 2–7)^†^	15.0 (9.0, 18.0)	10.5 (4.0, 15.8)
GFQ	24.0 (10.3, 33.5)	17.0 (7.0, 28.0)
LEDD, mg	823.8 ± 278.0	602.0 ± 235.2
MDS-UPDRS III, med-off	54.0 ± 12.0	—
MDS-UPDRS III, med-on	23.3 ± 9.1	—
Levodopa response, % improvement	57.28 ± 12.85	—
MDS-UPDRS III, DBS-off med-off	—	50.7 ± 13.7
MDS-UPDRS III, DBS-on med-off	—	29.0 (23.5, 43.0)
Stimulation response, % improvement	—	31.82 (21.67, 45.20)
Amplitude, V (left/right)	—	3.28/3.04
Frequency, Hz (left/right)	—	152/152
Pulse width, μs (left/right)	—	84/82

Normal data are expressed as mean ± standard deviation; non-normal data (Shapiro–Wilk *p* < 0.05) are expressed as median (lower quartile, upper quartile). GFQ baseline is reported as median (Q1, Q3) for consistency with its non-normal follow-up value, notwithstanding its own normal distribution (Shapiro–Wilk *p* = 0.156).

GFQ, Gait and Falls Questionnaire (16 items); FoGQ, Freezing of Gait Questionnaire; LEDD, levodopa equivalent daily dose; MMSE, mini-mental state examination; TD, tremor-dominant; ID, indeterminate; PIGD, postural instability and gait difficulty (motor phenotype defined by the ratio of mean tremor score to mean postural instability/gait difficulty score: TD ratio ≥1.15, PIGD ratio ≤ 0.90, ID otherwise; Stebbins et al., 2013).

^†^The FoGQ is the 6-item freezing subset of the GFQ (items 2–7); it is shown for comparability with prior studies and was not analyzed as a separate outcome. Levodopa response and stimulation response, % improvement are each computed within each individual from the corresponding MDS-UPDRS III scores as (med-off score–med-on or DBS-on med-off score)/med-off score × 100%, then summarized across participants (mean ± SD for the levodopa response, which was normally distributed; median [Q1, Q3] for the stimulation response, which was not). Stimulation parameters are follow-up values.

### Improvement of MDS-UPDRS III total score and its subitems by GPi-DBS

All four MDS-UPDRS III components—tremor, rigidity, bradykinesia and axial—improved significantly under the DBS-on med-off condition after GPi-DBS compared with the pre-operative med-off condition (Table 2). Within the axial component, the gait (3.10), freezing-of-gait (3.11), arising-from-chair (3.9) and posture (3.13) items each improved, whereas postural stability (3.12) did not; per-condition summaries and exact *p* values are given in Table 2. The per-item comparisons were exploratory; after FDR correction across the 10 items, the four summed components and the gait, posture, freezing-of-gait and arising-from-chair items remained significant, whereas the speech item (3.1) no longer reached significance (raw *p* = 0.048, FDR = 0.053). Overall, axial symptoms improved after the surgical intervention.

**Table 2:** MDS-UPDRS III components, including the individual items for the Axial component, pre-operative med-off vs. DBS-on med-off (*n* = 46).

MDS-UPDRS III	Preop med-off	DBS-on med-off	*p*	FDR	Sig.
Tremor component	6.50 (2.00, 14.75)	0.00 (0.00, 2.00)	< 0.001	< 0.001	Yes
Rigidity component	10.00 (8.00, 11.75)	6.50 (5.00, 9.75)	< 0.001	< 0.001	Yes
Bradykinesia component	25.00 (21.00, 31.00)	16.50 (12.00, 22.75)	< 0.001	< 0.001	Yes
Axial component	9.00 (6.25, 12.75)	6.00 (4.00, 9.00)	< 0.001	< 0.001	Yes
Speech (3.1)	1.00 (1.00, 2.00)	1.00 (1.00, 1.75)	0.048	0.053	No
Arising from chair (3.9)	1.00 (0.00, 1.00)	0.00 (0.00, 1.00)	0.011	0.014	Yes
Gait (3.10)	2.00 (1.00, 2.00)	1.00 (1.00, 2.00)	< 0.001	< 0.001	Yes
Freezing of gait (3.11)	1.50 (0.00, 2.00)	0.00 (0.00, 2.00)	0.001	0.002	Yes
Postural stability (3.12)	2.00 (1.00, 4.00)	2.00 (1.00, 3.00)	0.132	0.132	No
Posture (3.13)	1.50 (1.00, 3.00)	1.00 (0.00, 2.00)	< 0.001	< 0.001	Yes

Values are median (lower quartile, upper quartile); the MDS-UPDRS III is an ordinal scale and most components departed from normality. *p* by paired Wilcoxon signed-rank test; FDR = Benjamini–Hochberg across the 10 comparisons. After correction, the speech item (3.1) no longer reached significance.

### The effect of GPi-DBS on FoG at follow-up

Five objective indices in the TUG test (TUG total time, FoG total time, FoG episodes, proportion of FoG and the longest FoG duration) were compared with paired Wilcoxon signed-rank tests across the pre-specified contrasts (Figure 1, Table 3). For the primary contrast (pre-operative med-off vs. DBS-on med-off; *n* = 34), TUG total time decreased within individuals by a median of 5.0 s (95% CI 1.5–14.5; FDR = 0.003) and the longest FoG duration by 1.0 s (95% CI 0–3.5; FDR = 0.030). FoG total time, FoG episodes and FoG proportion changed in the same direction but did not survive FDR correction (FDR 0.064–0.080; Table 3). Because the distributions were right-skewed and one participant had a 687-s pre-operative TUG, values are summarized as median (Q1, Q3); repeating the primary contrast without this participant changed the median TUG-total reduction only from 5.0 to 4.0 s and it remained significant.

**Figure 1:**
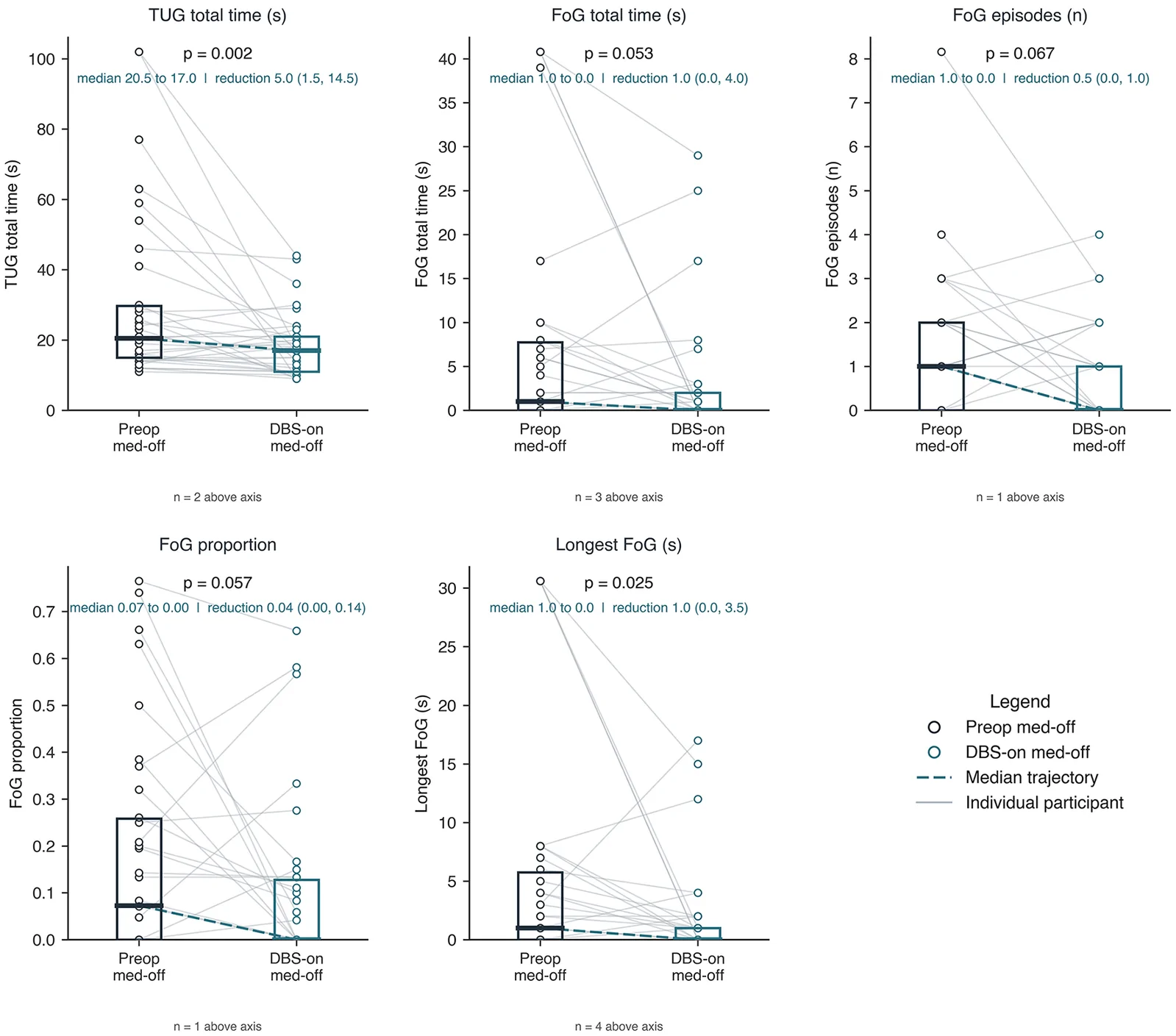
Within-individual change in the five Timed Up and Go (TUG) freezing-of-gait indices for the primary contrast (pre-operative med-off vs. DBS-on med-off; *n* = 34). Each thin gray line connects one participant's two values (pre-operative med-off, left; DBS- on med-off, right). For each condition, the open box shows the interquartile range, and the horizontal bar shows the median, and the dashed line joins the two medians. Printed in each panel are the exact *p* values (paired Wilcoxon signed-rank test), the two medians, and the within-individual (Hodges–Lehmann) median reduction with its 95% CI; participants whose values exceed the displayed axis are indicated (“*n* above axis”). A decrease denotes improvement.

**Table 3:** TUG freezing-of-gait indices by condition (*n* = 34).

Index, median (Q1, Q3)	Preop med-off	Preop med-on	DBS-off med-off	DBS-on med-off	Primary reduction^*^, HL (95% CI)	FDR^*^
TUG total time (s)	20.5 (15.0, 29.8)	12.0 (10.0, 13.8)	18.0 (15.0, 25.8)	17.0 (11.0, 21.0)	5.0 (1.5, 14.5)	0.003
FoG total time (s)	1.0 (0.0, 7.8)	0.0 (0.0, 0.0)	0.5 (0.0, 5.0)	0.0 (0.0, 2.0)	1.0 (0.0, 4.0)	0.064
FoG episodes (*n*)	1.0 (0.0, 2.0)	0.0 (0.0, 0.0)	0.5 (0.0, 2.0)	0.0 (0.0, 1.0)	0.5 (0.0, 1.0)	0.080
FoG proportion	0.07 (0.00, 0.26)	0.00 (0.00, 0.00)	0.02 (0.00, 0.21)	0.00 (0.00, 0.13)	0.04 (0.00, 0.14)	0.068
Longest FoG (s)	1.0 (0.0, 5.8)	0.0 (0.0, 0.0)	0.5 (0.0, 4.0)	0.0 (0.0, 1.0)	1.0 (0.0, 3.5)	0.030

Values are median (lower quartile, upper quartile); *n* = 34 (participants with all four conditions).

^*^Primary contrast = pre-operative med-off –DBS-on med-off (positive = improvement), Hodges–Lehmann median with distribution-free 95% CI; FDR over the six pairwise contrasts per index (paired Wilcoxon signed-rank).

No FoG-related TUG measure differed between the pre-operative med-off and DBS-off med-off conditions (all FDR ≥ 0.168); thus there was no evidence that the disease state was progressing over the short-term follow-up, consistent with this contrast's pre-specified role as a within-subject negative control rather than a basis for primary inference. The improvement observed with stimulation (DBS-on med-off vs. pre-operative med-off) is therefore not readily attributable to disease progression over this interval, although a genuine stimulation effect cannot be formally distinguished from other time-related factors in this single-arm design. Besides the assessed TUG performance, the total score of the self-reported GFQ decreased from a baseline median of 24 to 17 at follow-up (within-individual Hodges–Lehmann median difference 4.0, 95% CI 0–8; *p* = 0.036; Figure 2, Supplementary Table 3; Table 1). In an exploratory, FDR-corrected item-level analysis, only item 5 (duration of the longest freezing episode) remained significant across the 16 items (FDR = 0.035); items 2–4, significant only before correction, are shown descriptively in Figure 2B, and the GFQ conclusion is based on the total score. In a sensitivity analysis restricted to the 33 baseline- FoG-positive participants, the within-individual median GFQ reduction was similar (Hodges–Lehmann 4.0) but did not reach significance (*p* = 0.133), consistent with the smaller subgroup.

**Figure 2:**
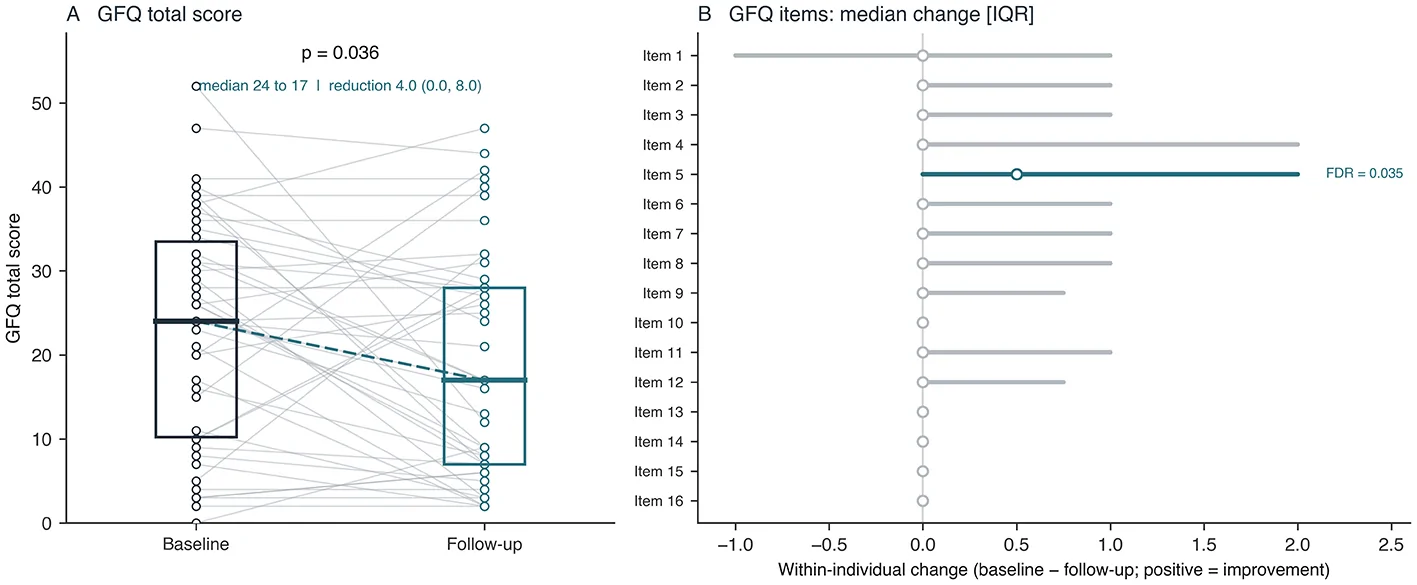
Within-individual change in the Gait and Falls Questionnaire (GFQ; *n* = 46). **(A)** GFQ total score at baseline **(left)** and follow-up **(right)**; each thin gray line is one participant, the open box shows the interquartile range with the median bar, and the dashed line joins the two medians. The exact *p* value (paired Wilcoxon signed-rank test), the median values, and *n* are printed. **(B)** Within-individual change (baseline minus follow- up; positive = improvement) for each of the 16 GFQ items, shown as the median (dot) and interquartile range (bar); after FDR correction across the 16 items, only item 5 (duration of the longest freezing episode) survived (labeled FDR < 0.05).

### Pre-operative axial levodopa-responsiveness is associated with post-operative FoG improvement

Forward stepwise AIC regression analysis identified the axial levodopa response (the difference of axial symptoms in MDS-UPDRS III between pre-operative med-off and med-on) as the sole pre-operative variable retained for all five indices of TUG change between pre-operative med-off and DBS-on med-off (Table 4), including TUG total time (β = 14.70, *p* = 0.008)—i.e., each 1-point greater pre-operative axial levodopa response (a 1-point larger reduction in the axial MDS-UPDRS III subscore from med-off to med-on) was associated with a 14.70-s greater within-individual improvement in TUG total time —, FoG total time (β = 13.87, *p* = 0.009), FoG episodes (β = 0.46, *p* = 0.004), the proportion of FoG (β = 0.04, *p* < 0.001) and the longest FoG duration (β = 7.61, *p* = 0.002); all five remained significant after FDR correction.

**Table 4:** Forward-stepwise AIC regression: pre-operative axial levodopa response predicting within-individual TUG change (*n* = 40).

Dependent variable (TUG change)	β	95% CI	*p*	FDR
TUG total time (s)	14.70	4.01, 25.40	0.008	0.009
FoG total time (s)	13.87	3.69, 24.05	0.009	0.009
FoG episodes (*n*)	0.46	0.16, 0.77	0.004	0.007
FoG proportion	0.04	0.02, 0.06	< 0.001	0.005
Longest FoG (s)	7.61	2.92, 12.31	0.002	0.005

Dependent variable = within-individual TUG change (pre-operative med-off–DBS-on med- off). Predictor = pre-operative axial levodopa response (med-off–med-on difference in the MDS-UPDRS III axial subscore). FDR added across the five models; all remain significant. *n* = 40 (participants with a complete pre-operative med-off and DBS-on med-off TUG pair; six of the 46 lacked one of these TUG assessments and were excluded).

We further conducted Spearman correlation analysis (Supplementary Table 4) to identify which motor component tracked gait improvement. Among the four motor components, the DBS- related change in the axial subscore was the only one correlated with within-individual TUG improvement, and this correlation survived FDR correction only for TUG total time (Spearman ρ = 0.431, 95% CI 0.138–0.654; *p* = 0.006, FDR = 0.028; *n* = 40). The axial correlations with FoG episodes (rho = 0.340, *p* = 0.032) and FoG total time (rho = 0.279, *p* = 0.082) were positive but did not survive FDR correction (FDR = 0.080 and 0.136), while tremor, rigidity and bradykinesia were not correlated, indicating an axial-specific association.

## Discussion

While the role of STN-DBS in PD motor symptom control has been extensively studied, the therapeutic potential of GPi-DBS, particularly for axial dysfunction and FoG, remains underexplored. Our present study provides evidence consistent with a post-operative reduction in gait-related deficits following GPi-DBS and explores its potential relevance for FoG, offering insights that may inform patient selection and outcome prediction. Over short-term follow-up, we observed improvements in FoG as reflected in both patient self-reported GFQ scores and video- assessed TUG performance with assessor blinding. Additionally, we found that pre-operative levodopa responsiveness of the axial component in MDS-UPDRS III was uniquely and positively associated with improvement in each of the five TUG indices.

Our study showed improvement in all MDS-UPDRS III subitems (tremor, rigidity, bradykinesia, and axial symptoms) under the DBS-on med-off condition at follow-up compared to pre-operative med-off state, with particularly notable benefits in axial symptoms, aligning with prior studies (Lee et al., 2022; Mei et al., 2020). The GPi projects through the pallidothalamic tracts (ansa and fasciculus lenticularis, converging in Forel's field H) to the ventral thalamus and brainstem, with indirect influence on the mesencephalic locomotor region and pedunculopontine nucleus; the gait changes we observed may therefore reflect modulation of these downstream locomotor pathways in addition to direct effects on pallidal motor circuitry.

Among the GFQ items, those relating to freezing—items 2 (walking difficulty in the worst state), 3 (impact of gait impairment on daily activities), 4 (daily FoG frequency) and 5 (longest single FoG episode duration)—improved after GPi-DBS, although only item 5 survived FDR correction and items 2–4 are shown descriptively (Figure 2B). These freezing items correspond to the six-item Freezing of Gait Questionnaire (FoGQ) (Bloem et al., 2016; Giladi et al., 2000), the subset of the GFQ (items 2 to 7) most widely used in prior studies; the FoGQ subscore [15.0 (9.0, 18.0) at baseline; 10.5 (4.0, 15.8) at follow-up; Table 1] is provided only for comparability with that literature, and because it is a strict subset of the GFQ it was not analyzed as a separate outcome. GFQ is more comprehensive than the FoGQ. While the FoGQ, derived from items 2 to 7 of the GFQ, primarily focuses on assessing the severity and characteristics of FoG itself, the GFQ's 16 items cover a broader range, including FoG, falls, festinating gait, and their impact on daily activities, thus providing a holistic view of gait-related disability and safety risks, which is critical for PD management. However, both scales have limitations. The FoGQ, as a shortened version focusing on FoG, fails to capture information related to falls and festinating gait. Meanwhile, neither the GFQ nor the FoGQ adequately assesses FoG in specific contexts such as dual-tasking, and they do not systematically account for daytime fluctuations in symptoms, which are important aspects of FoG assessment (Mancini et al., 2019). Despite this, the observed improvements in these freezing-related items, particularly in worst-state walking ability, are consistent with better gait continuity; quality of life and fall incidence were not measured here and remain to be assessed.

We then focused on comparisons between the pre-operative med-off condition and post-operative DBS-on med-off condition, which isolates the specific impact of DBS by eliminating medication effects through matched med-off states. Though a 1-h DBS shutdown cannot fully reverse stimulation benefits—especially slow-resolving axial effects (Cooper et al., 2013; Koeglsperger et al., 2019; Temperli et al., 2003)—this duration is the maximum patients can reliably tolerate and the ethical washout window in DBS trials. Supplementary pre-operative med-off vs. post-operative DBS-off med-off analyses yielded no significant differences; as in the Results, this is consistent with the primary contrast reflecting DBS-related effects rather than disease progression, though it does not on its own confirm this.

In terms of FoG outcomes, the TUG total time and longest FoG duration showed significant improvement in the DBS-on med-off condition, consistent with an association between GPi- DBS and reduced FoG severity and duration compared to the pre-operative med-off baseline. FoG total time, episodes, and proportion in TUG changed in the same direction but did not survive FDR correction (FDR 0.064–0.080); these differences are therefore inconclusive and require validation in larger cohorts or with extended follow-up.

Our study is consistent with a post-operative effect of GPi-DBS on FoG, examined through clinical phenotypic factors. To identify pre-operative predictors of FoG outcomes, we employed a forward stepwise AIC regression model incorporating 28 variables (pre-operative demographic and clinical variables). Compared to traditional generalized linear models, this approach avoids collinearity and refines key factors by balancing model complexity and goodness of fit. In our analysis, the pre-operative axial levodopa response was the only pre-operative variable retained by the model, suggesting a relationship with subsequent TUG improvement that warrants confirmation in independent samples. Furthermore, our Spearman correlation analysis revealed no significant association between post-operative FoG improvement and DBS-related changes in bradykinesia, tremor, or rigidity—highlighting the axial-specific association with FoG response to GPi-DBS. This axial specificity is consistent with the dopaminergic phenotype described by McKay et al. (2019). Using an acute supratherapeutic levodopa challenge comparable to ours, they found that among patients whose freezing persisted in the full ON state, other axial features were likewise levodopa-unresponsive, whereas the appendicular domains responded similarly across groups. Axial signs and freezing therefore appear to share a common dopaminergic responsiveness that is dissociable from that of the appendicular (limb) domains, which offers a rationale for why the pre-operative axial levodopa response—rather than the tremor, rigidity or bradykinesia response—tracked the subsequent gait outcome in our cohort.

In contrast, Lee et al. (2022) conducted a pilot study on 20 PD patients undergoing GPi- DBS. They classified patients into FoG responders and non-responders. Their study used logistic regression, focusing on anatomical and functional imaging data. The treatment correlates identified included disease duration, pre-operative levodopa responsiveness of FoG, and the proximity of active DBS contacts to the prefrontal subregion of the GPi. In terms of pre-operative indicators, while we concentrated on demographic and clinical parameters, they delved into factors related to DBS electrode placement. Additionally, they explored potential biological mechanisms such as prefrontal GPi connectivity and modulation of locomotor networks. Together, these different approaches underscore the multifactorial nature of treatment outcomes in PD patients with FoG. Integrating multimodal assessments, such as combining our clinical- based approach with the imaging-based insights from studies like Lee et al. (2022)'s, may enhance the predictive precision in the management of FoG with GPi-DBS.

Our study has unique strengths. First, we analyzed the presence and severity of FoG not only in a subjective manner by incorporating patients' self-reported specific GFQ but also in a video-based manner by assessing quantitatively the TUG test with assessor blinding. These methodological enhancements contribute to the robustness and specificity of our findings, compared to the previous research (Hwang et al., 2023). FoG, characterized by its heterogeneous and episodic nature, is often underdetected during brief clinic assessments. The GFQ scale, however, captures patients' self-reported severity of FoG across daily contexts, providing a more comprehensive reflection of its insidious impact. Meanwhile, the TUG test offers granular objectivity by decomposing gait phases, which is critical given the multifaceted pathophysiology of FoG. We classified freezing by its response to an acute suprathreshold levodopa challenge; because such a challenge achieves adequate dopaminergic stimulation, freezing that persisted (the partially levodopa-responsive and levodopa-unresponsive strata) reflects genuine dopaminergic resistance rather than an under-dosed pseudo-ON state (Zibetti et al., 2018), in keeping with the distinction between levodopa-responsive (OFF) freezing and freezing that persists after an acute levodopa challenge (McKay et al., 2019), which builds on the classical subtyping of off- vs. on-state freezing by its levodopa response (Schaafsma et al., 2003) and the recognition of genuine on-state, dopa-resistant freezing (Espay et al., 2012). While disentangling the neural circuits underlying each FoG subtype remains challenging, our detailed TUG subitem analysis could provide pivotal clues for future mechanistic studies. This “phenomenon-driven mechanism exploration” framework may bridge the gap between traditional circuit models and the complexity of gait symptomatology. Furthermore, the inclusion of a larger sample size in this area strengthens the robustness of our findings. The cohort spanned a range of disease severity, which aids generalizability but limits subgroup precision. Second, the study was designed in a video ratings-blinded, prospective manner, instead of retrospective chart reviews as in other long-term studies, to assess both quantitative and subjective measures.

Advancements in imaging and neurophysiological techniques may further elucidate these mechanisms, and functional MRI findings in PD with FoG have been systematically reviewed (Song et al., 2021). For instance, a functional MRI study showed that activity in the right GPi and left middle occipital gyrus was significantly reduced in PD patients with FoG, and this reduction correlated with GFQ and TUG performance (Kou et al., 2022). Another imaging study found that the right GPi was associated with levodopa responsiveness in FoG (Lench et al., 2022). These results could serve as speculative mechanistic explanations for our clinical findings, suggesting that modulating the GPi and its associated neural networks may correct aberrant signaling patterns contributing to FoG, thereby improving gait performance. Continued research is vital for optimizing its application and understanding the complex mechanisms underlying its effects.

A small subset of patients (8 of 46) showed worsening of self-reported freezing (an increase in MDS-UPDRS II item 2.13 from baseline to final follow-up) at follow-up. In an exploratory comparison, these patients had, if anything, the smallest reduction in dopaminergic medication (median 25 vs. 242 mg) and somewhat poorer baseline cognition (MMSE 26.9 vs. 27.6; Supplementary Table 5), rather than the aggressive levodopa withdrawal often invoked to explain post-DBS gait deterioration. This pattern is consistent with the view that, in a subgroup of patients, freezing is driven pre-dominantly by non- dopaminergic, non-pallidal mechanisms—cholinergic denervation of the pedunculopontine nucleus and mesencephalic locomotor region (Bohnen et al., 2019; Karachi et al., 2010), together with frontal–executive and attentional network dysfunction (Amboni et al., 2008; Shine et al., 2013)—which are modulated neither by levodopa nor globus pallidus stimulation and progress with the disease. The worsening in these patients is therefore more consistent with progression of a cognitive–axial, treatment-refractory freezing phenotype than with a stimulation-induced effect, although our data cannot distinguish the two. These observations are exploratory (*n* = 8) and hypothesis-generating.

Nevertheless, our study has limitations. First, as a single-arm observational study without randomization or a direct STN comparator, the findings are associations and cannot establish causal or comparative superiority. Second, follow-up was short-term and of variable duration (median 11 months, range 6–34); this window may be insufficient to capture the long-term effects of GPi-DBS on FoG, which may attenuate as the disease advances; a longer-term, protocol-timed follow-up of this cohort is planned. Third, multicenter replication would improve external validity and would increase the sample size, giving more statistical power. The absence of provocative maneuvers (e.g., turning, dual-task walking) may have underdetected brief FoG episodes. The FoG subgroups were heterogeneous, and the partially responsive (*n* = 7) and unresponsive (*n* = 3) strata are too small for inferential subgroup testing. Additionally, the lack of neuroimaging or electrophysiological measures (task-specific fMRI, local field potential recordings, wearable inertial sensors, and intraoperative EEG) hinders direct exploration of the neural mechanisms underlying FoG improvement, and we make no mechanistic claims. Incomplete data on the DBS-on med-on condition precluded its inclusion. Finally, the outcome distributions were right-skewed and sensitive to extreme values, which is why the analysis is rank-based.

## Conclusion

In conclusion, the present study aimed to investigate the effect of GPi-DBS on FoG in PD patients and identify pre-operative predictors of such effect. Within this single-arm cohort, FoG measures improved over short-term follow-up after GPi-DBS, as evidenced by both self- reported questionnaire results and quantitative analysis of TUG test metrics. Importantly, greater pre-operative axial levodopa-responsiveness (assessed via MDS-UPDRS III) was associated with greater TUG improvement and may help identify candidates likely to benefit. Conversely, a small, poor cognitive–axial subgroup whose FoG worsened appeared not to benefit; this observation is exploratory (*n* = 8, no comparison significant), and whether such patients warrant strategies targeting non-dopaminergic mechanisms rather than pallidal stimulation alone requires confirmation. These results, derived from a relatively larger sample size and specialized assessment tools, describe associations rather than causal effects; controlled studies with longer follow-up and imaging are needed to test causality, durability and mechanism.

## Data Availability

The raw data supporting the conclusions of this article will be made available by the authors, without undue reservation.
