# Supplementary material for: Effects of globus pallidus internus deep brain stimulation on freezing of gait in Parkinson's disease: a prospective short-term single-arm study
Source: Front Aging Neurosci. 2026 Sep 28;18:1848040. doi: 10.3389/fnagi.2026.1848040 (PMC13655047; doi:10.3389/fnagi.2026.1848040)
Supplement: Supplementary file 1 [file Data_Sheet_1.docx]

Supplementary Table 1. Descriptive change by FoG subtype (exploratory).

| FoG subtype | n | GFQ total change [IQR] | TUG total change (s) [IQR] | Longest FoG change (s) [IQR] |
| --- | --- | --- | --- | --- |
| OFF-FoG | 23 | 5.0 [-5.5, 12.5] (n=23) | 6.0 [2.0, 27.0] (n=21) | 2.0 [0.0, 7.0] (n=21) |
| partially levodopa-responsive | 7 | 0.0 [-1.0, 3.0] (n=7) | 16.0 [1.5, 45.8] (n=4) | 6.0 [-0.5, 20.0] (n=4) |
| levodopa-unresponsive | 3 | 20.0 [10.0, 30.0] (n=3) | 11.5 [6.8, 16.2] (n=2) | 1.5 [0.8, 2.2] (n=2) |
| All FoG-positive | 33 | 3.0 [-2.0, 15.0] (n=33) | 6.0 [2.0, 28.5] (n=27) | 2.0 [0.0, 7.0] (n=27) |

Within-individual changes as median [IQR] (positive = improvement); GFQ = baseline − follow-up, TUG = preoperative med-off −DBS-on med-off. Strictly descriptive and exploratory; the strata are far too small (partially responsive n=7, unresponsive n=3) for meaningful subgroup inference, so no test is reported.

Supplementary Table 2. FoG status transition (MDS-UPDRS II item 13), baseline vs follow-up.

| Baseline status | Follow-up FoG-positive (≥1) | Follow-up FoG-negative (0) | Row total |
| --- | --- | --- | --- |
| FoG-positive (item 13 ≥ 1) | 26 | 7 | 33 |
| FoG-negative (item 13 = 0) | 2 | 11 | 13 |
| Column total | 28 | 18 | 46 |

FoG status by MDS-UPDRS II item 13 (positive = ≥1). Among baseline FoG-positive patients, self-reported severity decreased over the follow-up (Wilcoxon signed-rank p = 0.022); the binary prevalence (33/46 →28/46) did not change significantly (McNemar exact p = 0.180).

Supplementary Table 3. Gait and Falls Questionnaire — within-individual change (n = 46).

| GFQ measure | Baseline median (Q1, Q3) | Follow-up median (Q1, Q3) | Improvement from baseline [95% CI] | p |
| --- | --- | --- | --- | --- |
| Total score (16 items) | 24 [10–34] | 17 [7–28] | 4.0 (0.0, 8.0) | 0.036 |

Improvement from baseline = baseline −follow-up within each participant (positive = improvement); Hodges–Lehmann median with distribution-free 95% CI. Per-item exploratory comparisons were FDR-corrected across the 16 items; only item 5 (duration of the longest freezing episode) survived correction (FDR = 0.035). n = 46.

Supplementary Table 4. **Spearman correlations of DBS-related change in MDS-UPDRS III motor subscore (preoperative med-off − DBS-on med-off) and each of the 5 within-individual TUG changes; based on n = 40. Tremor, rigidity and bradykinesia correlations are summarized in the footnote.**

| **TUG change** | **ρ (95% CI)** | **FDR** |
| --- | --- | --- |
| TUG total time | **0.431 (0.138, 0.654)** | 0.028* |
| FoG total time | **0.279 (-0.036, 0.543)** | 0.136 |
| FoG episodes | **0.340 (0.031, 0.589)** | 0.080 |
| FoG proportion | **0.194 (-0.125, 0.477)** | 0.229 |
| Longest FoG | **0.233 (-0.084, 0.508)** | 0.184 |

Predictor = DBS-related change in each MDS-UPDRS III motor subscore (preoperative med-off −DBS-on med-off); outcome = corresponding within-individual TUG change. FDR = Benjamini–Hochberg across the five correlations; after correction, only the axial change × TUG-total-time association remains significant. Among the four motor **components**, only the axial change is correlated with TUG improvement (tremor r=-0.11; rigidity r=0.26; bradykinesia r=0.13). This DBS-related axial change is distinct from the preoperative axial levodopa response used in Table 4. n = 40; six of the 46 participants lacked either the preoperative or the DBS-on med-off TUG assessment and therefore could not contribute a within-individual TUG change, so they were excluded from this analysis. *FDR<0.05; **FDR<0.01.

Supplementary Table 5. Exploratory profile of patients whose FoG worsened.

| Variable | Worsened (n=8) mean (median) | Others (n=38) mean (median) | Mann–Whitney p |
| --- | --- | --- | --- |
| LEDD reduction, mg | 84 (25) | 251 (242) | 0.079 |
| MMSE | 26.9 (27) | 27.6 (28) | 0.057 |
| Age at onset, y | 54.5 (55) | 49.7 (51) | 0.147 |
| Disease duration, y | 11.8 (12) | 10.7 (10) | 0.432 |
| Baseline LEDD, mg | 682 (675) | 854 (850) | 0.124 |

FoG worsening = increase in MDS-UPDRS II item 13 from baseline to follow-up (n=8). Strictly exploratory; no difference survives multiple-comparison correction. Notably, the worseners had the smallest medication reduction, arguing against levodopa withdrawal.
